# Supplementary material for: GNN Codon Adjacency Tunes Protein Translation
Source: Int J Mol Sci. 2024 May 29;25(11):5914. doi: 10.3390/ijms25115914 (PMC11172435; doi:10.3390/ijms25115914)
Supplement: Supplementary file 1 [file ijms-25-05914-s001.zip › ijms-3031800-supplementary.pdf]

Supporting Information

GNN Codon Adjacency Tunes Protein Translation

Joyce Sun [1,†], Pete Hwang [1,†], Eric D. Sakkas [1], Yancheng Zhou [1], Luis Perez [1], Ishani Dave [1], Jack B. Kwon [1], Audrey E. McMahon [1], Mia Wichman [1], Mitsu Raval [1], Kristen Scopino [1], Daniel Krizanc [2,3], Kelly M. Thayer [2,3,4] and Michael P. Weir [1,3,*]

[1] Department of Biology, Wesleyan University, Middletown, CT 06459, USA; jsun02@wesleyan.edu (J.S.); phwang@wesleyan.edu (P.H.); esakkas@wesleyan.edu (E.D.S.); ezhou@wesleyan.edu (Y.Z.); lperez@wesleyan.edu (L.P.); idave@wesleyan.edu (I.D.); jkwon@sail.bio (J.B.K.); aemcmahon@wesleyan.edu (A.E.M.); mwichman@wesleyan.edu (M.W.); mraval@wesleyan.edu (M.R.)

[2] Department of Mathematics and Computer Science, Wesleyan University, Middletown, CT 06459, USA; dkrizanc@wesleyan.edu (D.K.); kthayer@wesleyan.edu (K.M.T.)

[3] College of Integrative Sciences, Wesleyan University, Middletown, CT 06459, USA

[4] Department of Chemistry, Wesleyan University, Middletown, CT 06459, USA

* Correspondence: mweir@wesleyan.edu; Tel.: +1-(860)-685-2402

† These authors contributed equally to this work.

Supplemental Figures and Tables

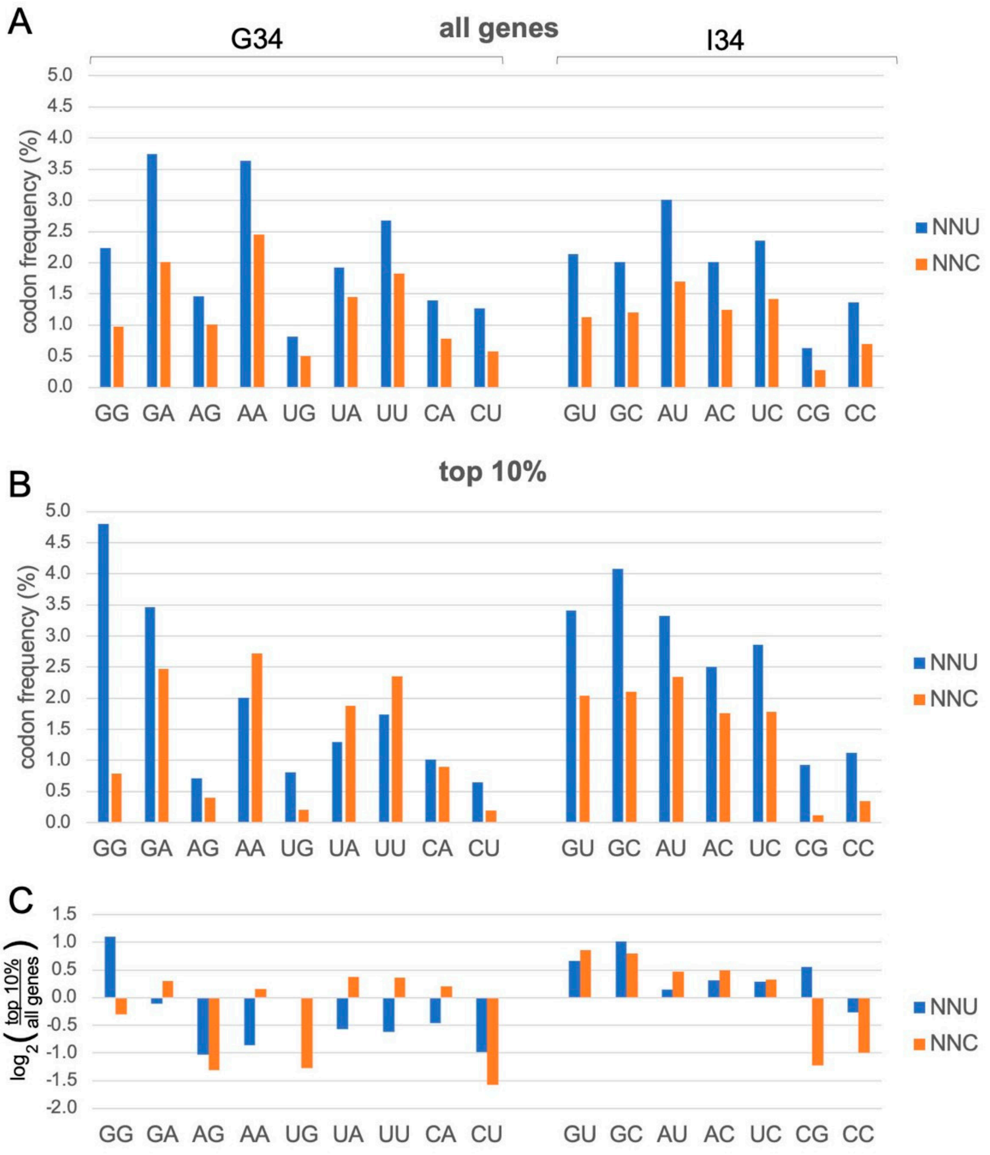

Figure S1. NNU/C codon frequencies. (A) For all NNU/C codon pairs, NNU codons have significantly higher frequencies in yeast ORFs than NNC (bootstrap $p < 0.01$). Codons are labeled on the x-axis according to their first two nucleotides. (B) Genes with high protein expression (top 10%, see below) show similar trends. NNU codons have higher frequencies than NNC for all NNU/C codon pairs that utilize tRNA I34 anticodons, and most NNU/C codon pairs that utilize G34, except for AAU/C, UAU/C and UUU/C where NNC codons have significantly higher frequencies (bootstrap $p < 0.01$). We extracted the top 10% of protein expressers from a set of 3868 genes with detectable protein expression in a genomic-scale western and reporter gene analysis in yeast [1]. (C) The NNU/C codon frequencies in high expression genes compared to all genes are significantly elevated for all codons that utilize I34 except CGC, CCC and CCU. In contrast, for codons that utilize G34, most NNU and some NNC codon frequencies are significantly depressed.

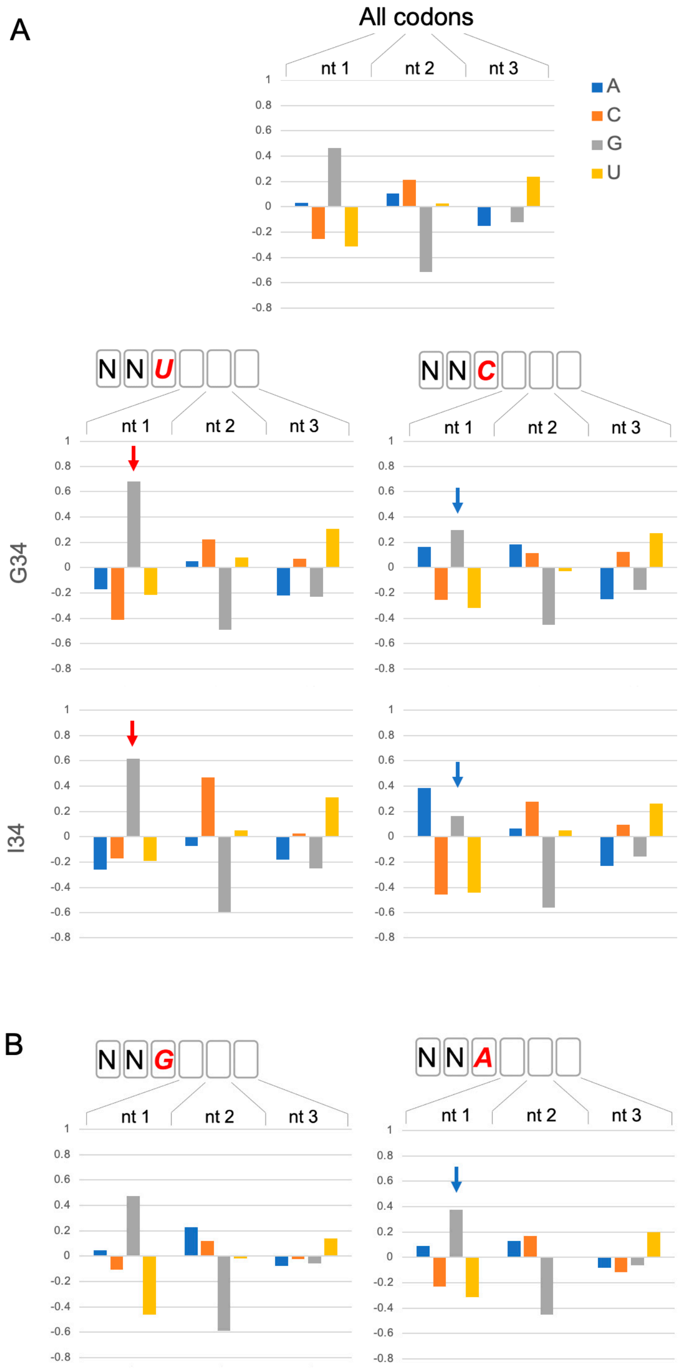

Figure S2. GNN codon adjacency in yeast. (A) Position weight matrices of yeast ORFs (6703 genes) show that codons following (3') NNU and NNC have elevated (red arrows) and depressed (blue arrows) GNN frequencies respectively, whether they utilize G34 or I34 tRNAs (bootstrap $p < 0.01$). (B) NNG codons do not show these trends; the codons following NNA have depressed GNN weights (bootstrap $p < 0.01$). Codon nucleotide preferences are illustrated by $\log_2(f_{obs}/f_{exp})$ using background (expected) frequencies of nucleotides in ORFs regardless of codon position (A:0.325; C: 0.193; G: 0.204; U:0.278).

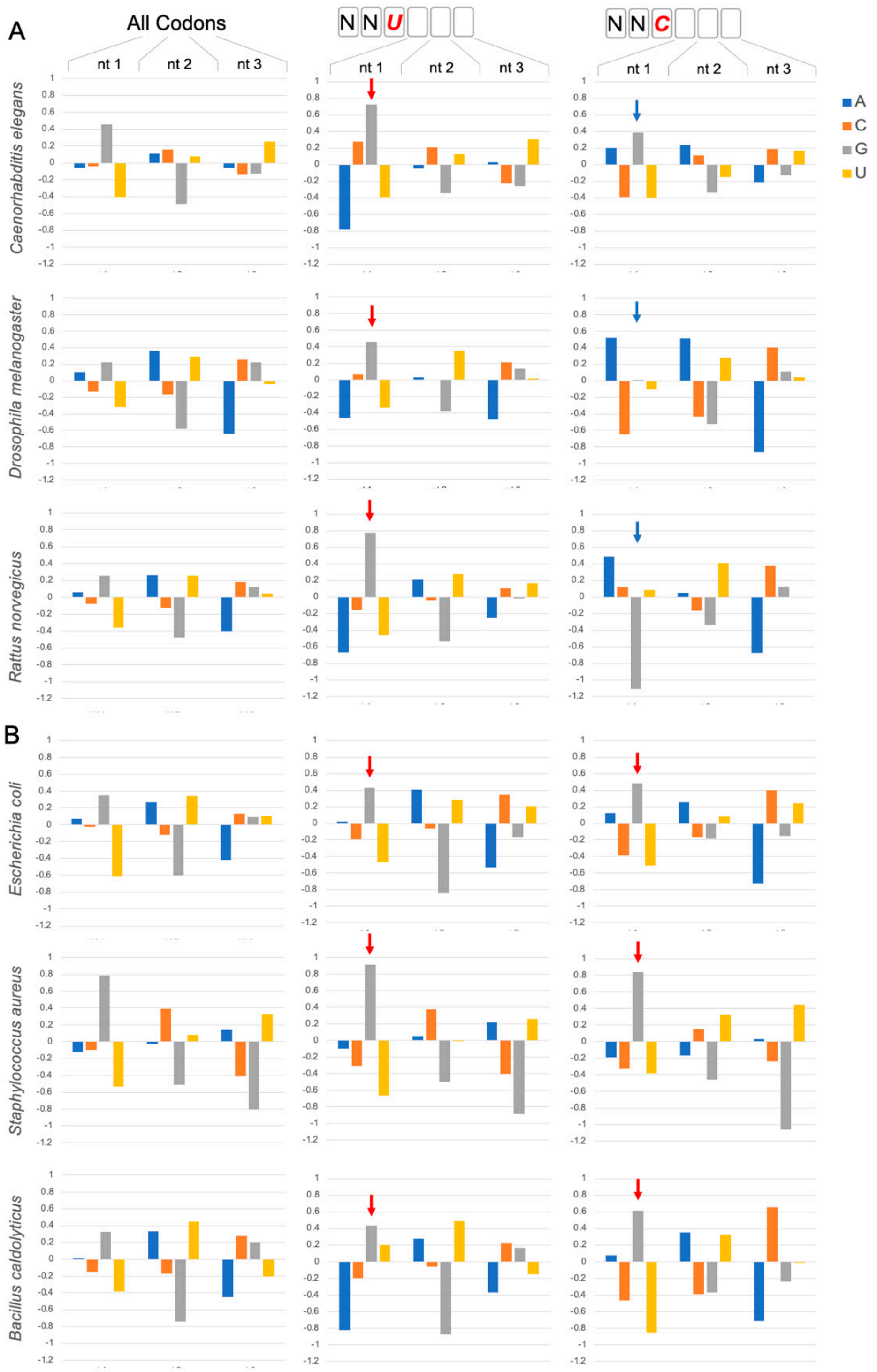

Figure S3. GNN codons are overrepresented in eukaryotes and prokaryotes. Position weight matrices illustrating $\log_2(f_{obs}/f_{exp})$ show that GNN codons are overrepresented in ORFs across multiple species. In the eukaryotes (A), GNN is significantly more abundant when 3' adjacent to NNU codons (bootstrap $p < 0.01$, red arrows) and GNN is significantly less abundant when adjacent to NNC codons (bootstrap $p < 0.01$, blue arrows). However, in the prokaryotes (B), GNN is significantly more abundant adjacent to NNU and NNC codons (bootstrap $p < 0.01$, red arrows).

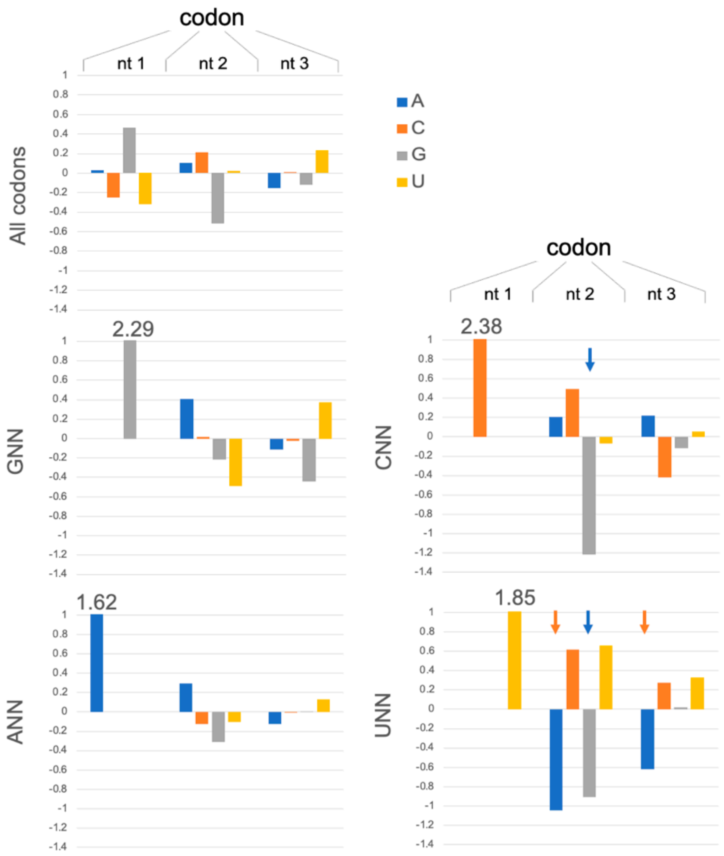

Figure S4. Codon nucleotide underrepresentation in different codon classes. Position weight matrices of yeast ORFs show that G2 (nt 2) underrepresentation in codons is particularly pronounced in CNN and UNN codons (blue arrows) and much weaker in GNN and ANN codons. The selection in UNN codons against G2 as well as A2 and A3 (orange arrows) is likely explained in part by selection against UGA, UAA and UAG stop codons. The selection against G2 in CNN codons may be explained in part by observations [2,3] that CGN codons have slow translation and that CGA codons are prevalent in inhibitory codon pairs that depress translation as well as observations [4] that CGA-containing inhibitory codon pairs in the ribosome's P and A sites appear to have structures that would interfere with translation. The values of position weights for the invariant nucleotides are labeled above their bars.

WT2: 28-nt RFPs

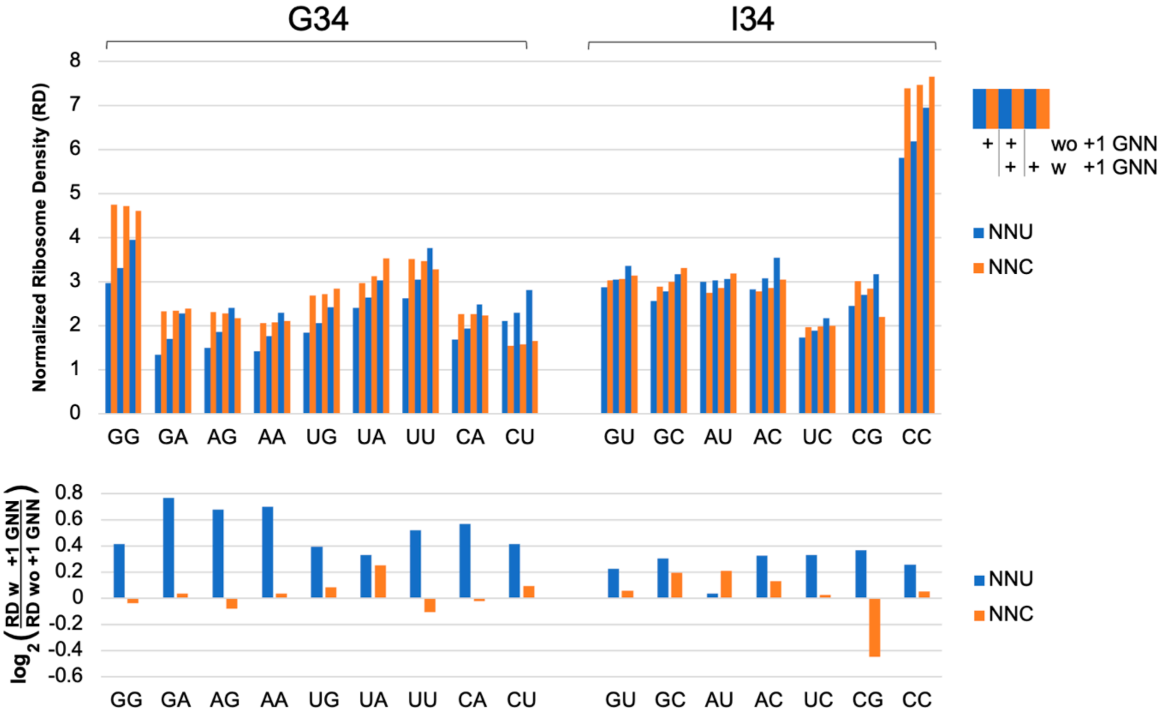

Figure S5. Independent replicate ribosome profile analysis of NNU/C codons. Equivalent analysis to Figure 2 but with independent (WT2) replicate dataset from the same study [5].

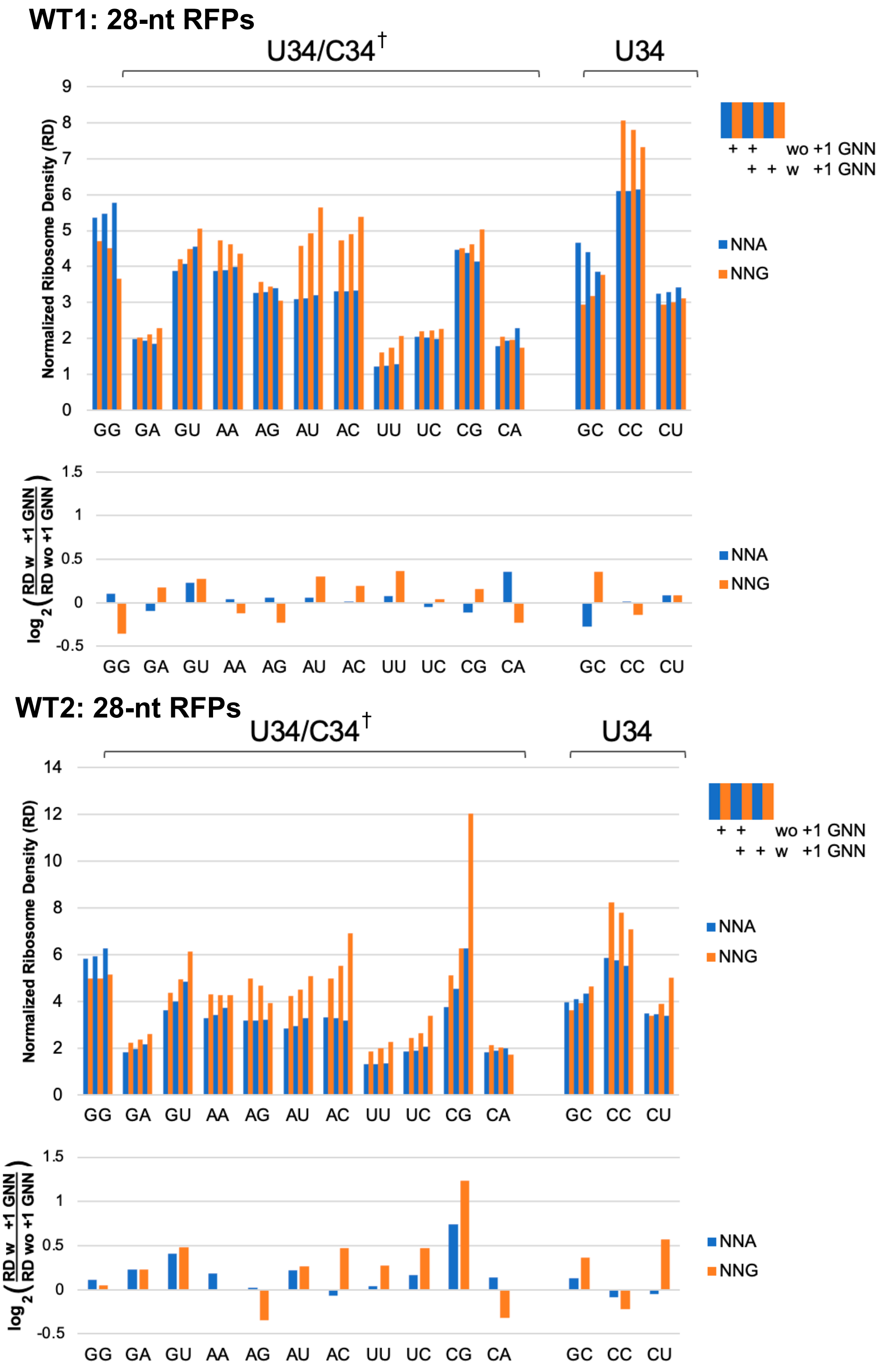

Figure S6. Ribosome profile analysis of NNG and NNA codons. Several A-site NNG (GCG, GAG, GUG, AUG and UUG) ribosome profiles (28-nt RFPs) had significantly increased densities when followed by +1 GNN in both replicate experiments [5] (WT1 and WT2; bootstrap $p < 0.01$; Table S1) whereas CAG and AGG had reduced densities. Only one NNA codon (GUA) had significantly increased densities with +1 GNN in both replicates. Data in Figures S5, S6, and S7 is graphed as in Figures 2 and S4. GGG/A, GAG/A and GUG/A codon pairs share the same U34 tRNA; the other NNG/A codon pairs have separate tRNAs for each codon. Note that CGA codons in yeast utilize I34 instead of U34 (†).

WT1: 21-nt RFPs

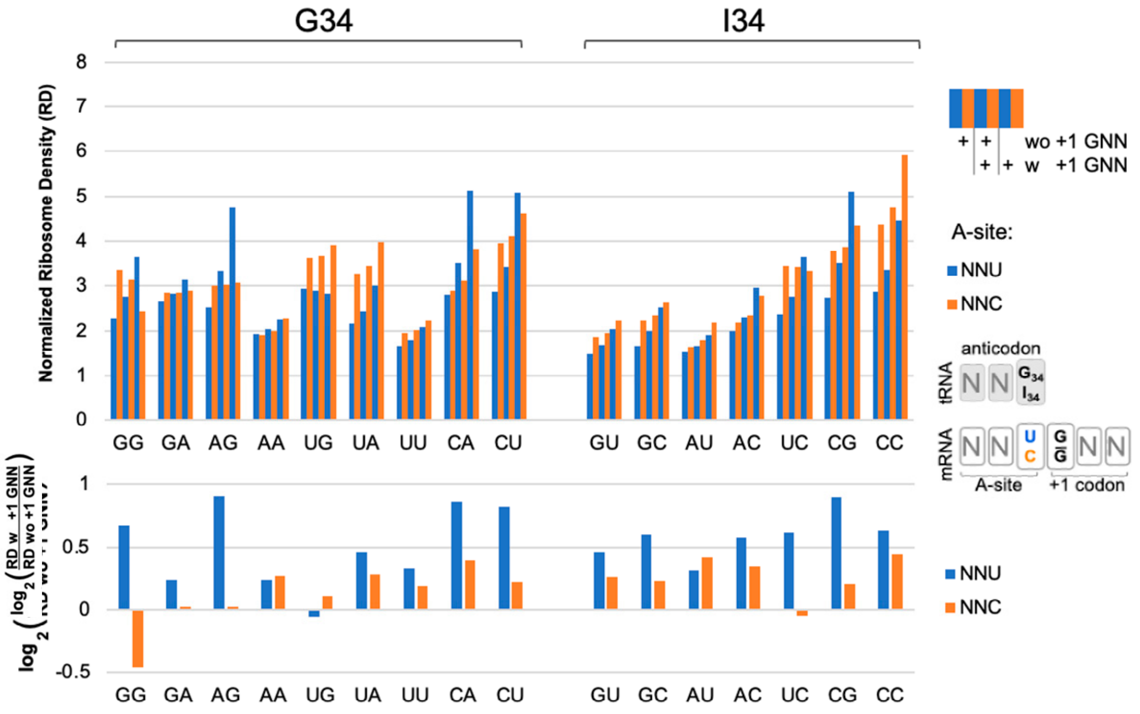

WT2: 21-nt RFPs

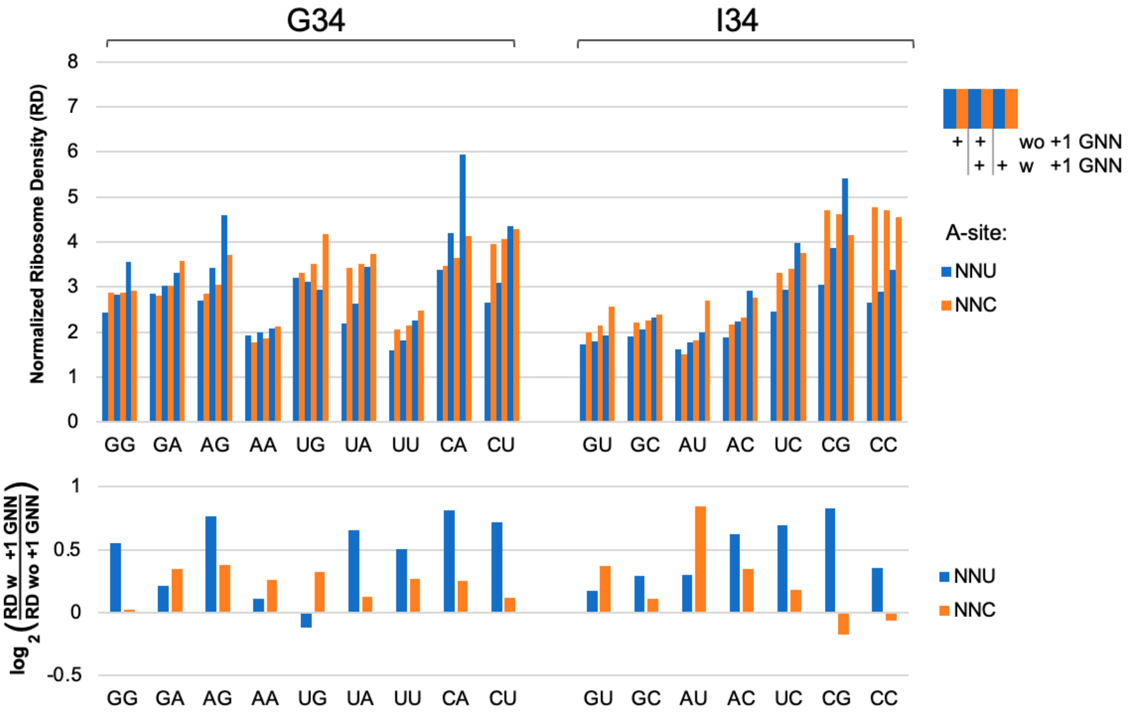

Figure S7. Pre-accommodation footprints. Ribosome footprints of length 20-22 nucleotides (21-nt RFPs) are hypothesized to represent pre-accommodation ribosomes that have not accommodated a tRNA at their A-site. The 21-nt RFPs showed similar trends to the larger 28-nt RFPs except that both A-site NNU and NNC codons show higher densities when followed by +1 GNN codon. WT1 and WT2 are independent replicates.

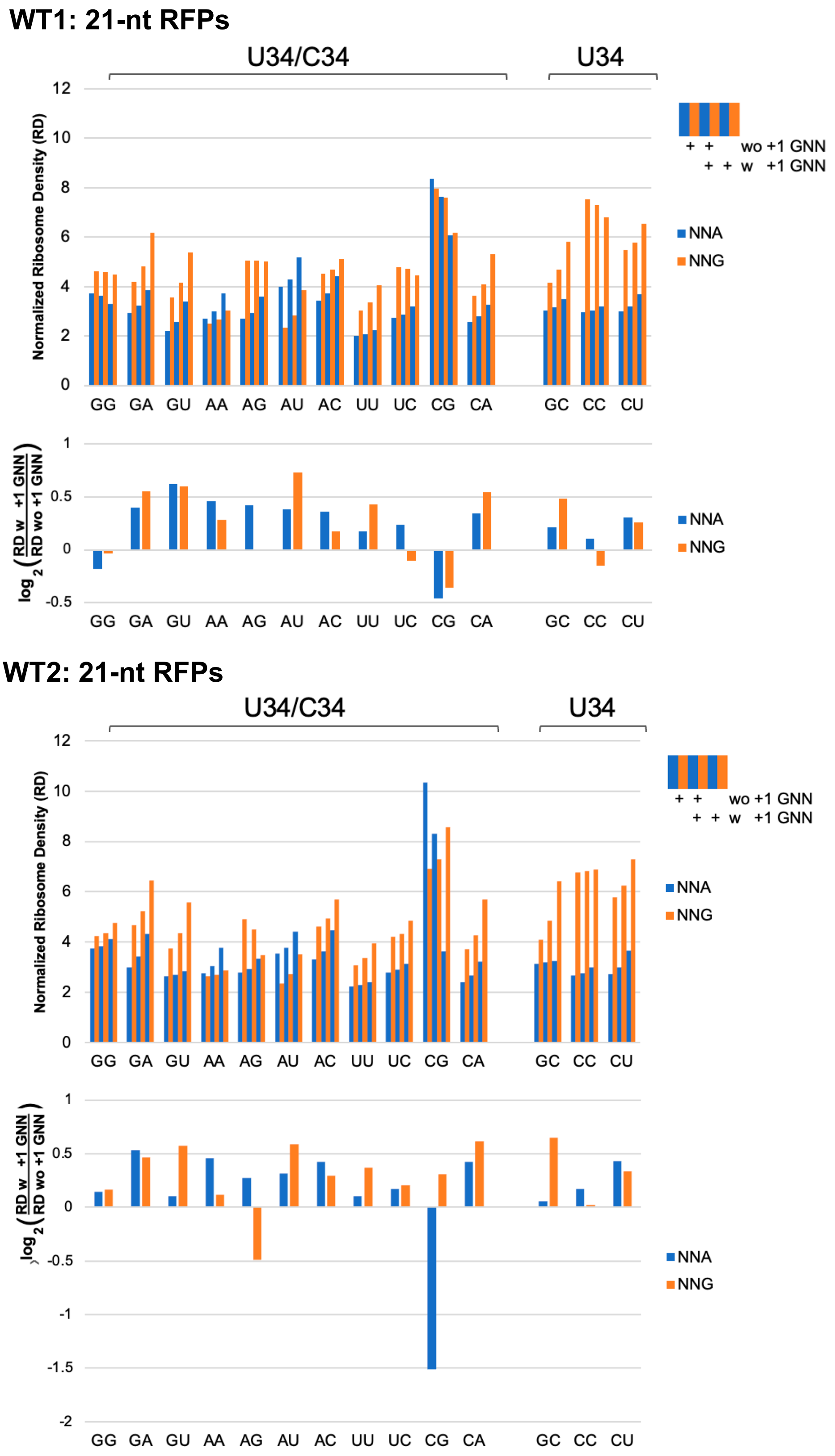

Figure S8. Pre-accommodation footprints. Many A-site NNA and NNG codons had elevated ribosome densities when followed by +1 GNN codons (see Table S2). Note that CGA RFPs (replicate WT2) had small numbers of CGA GNN codon pairs with above-threshold ribosome densities (>1 footprint per 10 nt).

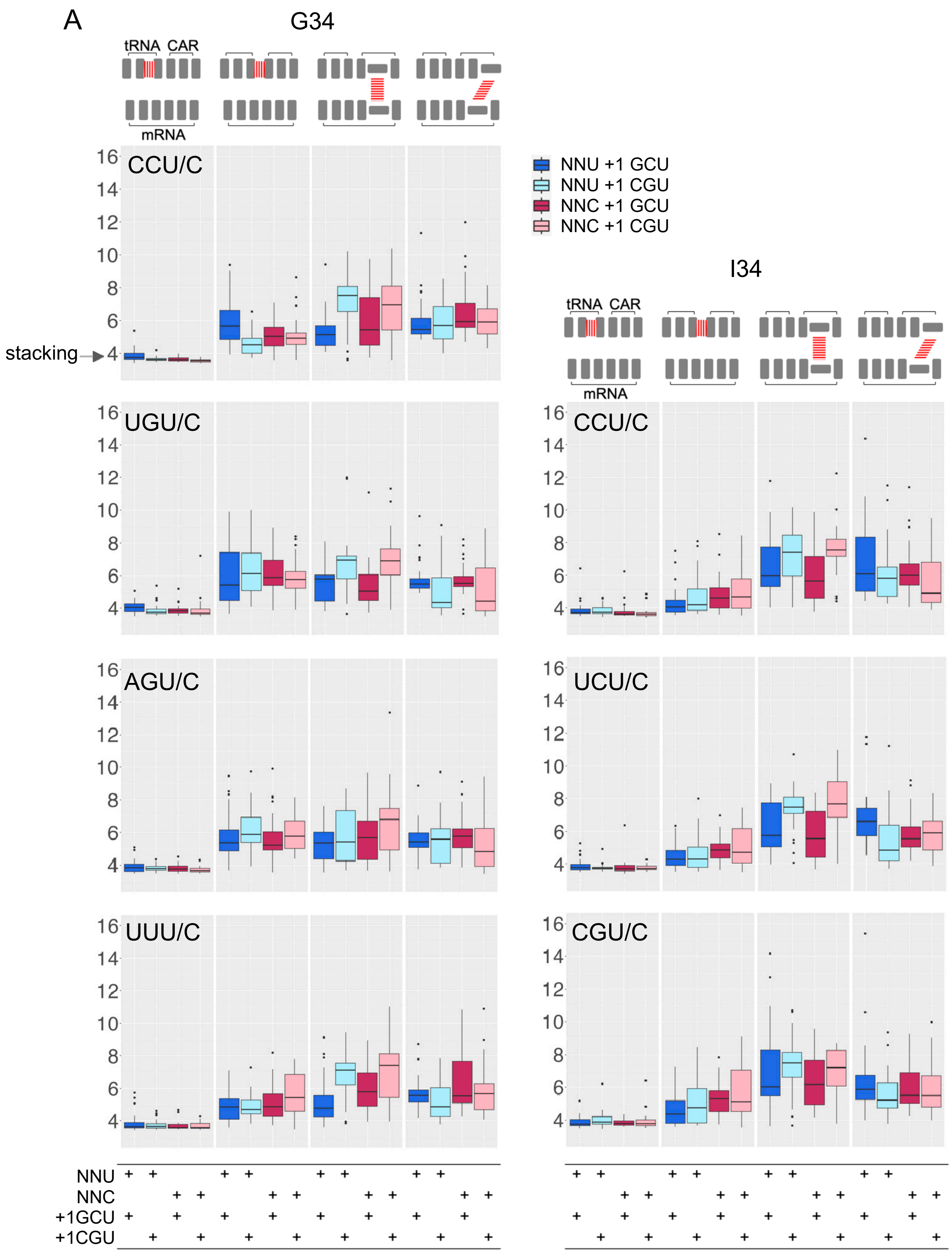
A
G34
tRNA CAR
mRNA
stacking
CCU/C
UGU/C
AGU/C
UUU/C
NNU +1 GCU
NNU +1 CGU
NNC +1 GCU
NNC +1 CGU
I34
tRNA CAR
mRNA
CCU/C
UCU/C
CGU/C
NNU
NNC
+1GCU
+1CGU

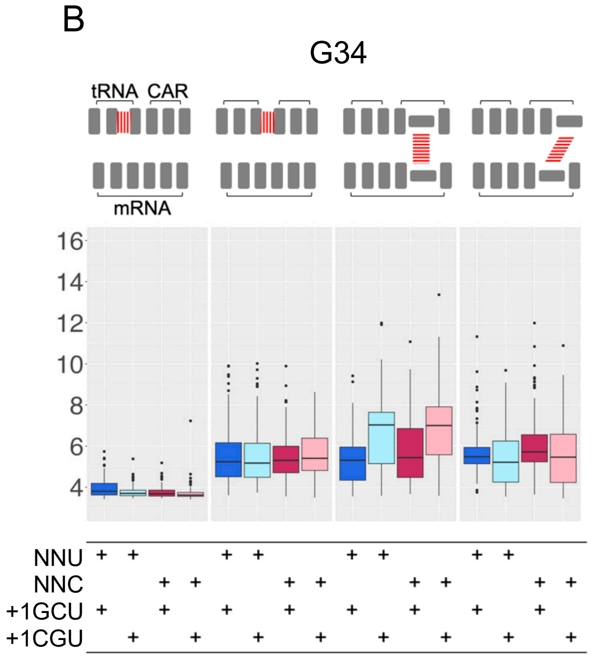

Figure S9. Stacking behavior of NNU/C codons. (A) Illustrated left to right (see top diagram) is pi-stacking between bases (tRNA nt35:nt34; nt34:C1054; A1196:+1 nt2) or pi-cation stacking between +1 nt2 and R146 (guanidinium group). Graphs are labeled according to the A-site NNU/C identities, and the NNU or NNC codons are immediately followed by +1 GCU or +1 CGU as summarized in the bottom table. 30 MD replicates were performed for each construct. Stacking was observed when the distances between the centers of geometry of base rings or the guanidinium group of R146 were within 4 Å which due to space constraints resulted in approximately parallel planes of the base rings (or guanidinium group). Nt34:nt35 showed consistent stacking distances of about 4 Å. A-site NNU/C with +1 GCU often showed stacking of A1196 with +1 nt2. A-site NNU/C with +1CGU often showed stacking of R146 with +1 nt2 (see Figure 4). Anchoring of CAR to tRNA nt34 (C1054:nt34) was particularly pronounced for codon pairs (CGU/C, UCU/C, CCU/C) that utilize I34. (B) Collation of data from part A for G34 (120 replicate MD experiments) and I34 (90 replicates) tRNAs.

Table S1: 28-nt RFPs bootstrap analysis (2-tailed; $p < 0.01$)

	+1GNN > wo +1GNN		+1GNN < wo +1GNN*	
	**WT1**	**WT2**	**WT1**	**WT2**
G34 NNUs				
GGU	H	H	-	-
GAU	H	H	-	-
AGU	H	H	-	-
AAU	H	H	-	-
UGU	H	H	-	-
UAU	H	H	-	-
UUU	H	H	-	-
CAU	H	H	-	-
CUU	H	H	-	-
I34 NNUs				
GUU	H	H	-	-
GCU	H	H	-	-
AUU	-	-	L	-
ACU	H	H	-	-
UCU	H	H	-	-
CGU	H	H	-	-
CCU	-	H	-	-
G34 NNCs				
GGC	-	-	-	-
GAC	-	-	L	-
AGC	-	-	-	-
AAC	-	-	L	-
UGC	-	-	-	-
UAC	-	H	-	-
UUC	-	-	-	-
CAC	-	-	-	-
CUC	-	-	-	-
I34 NNCs				
GUC	-	-	-	-
GCC	-	H	-	-
AUC	-	H	-	-
ACC	-	-	-	-
UCC	-	-	-	-
CGC	-	-	-	-
CCC	-	-	-	-

	+1GNN > wo +1GNN		+1GNN < wo +1GNN*	
	WT1	WT2	WT1	WT2
U34 NNAs				
GCA	-	-	L	-
CCA	-	-	-	-
CUA	-	-	-	-
U/C34 NNAs				
GGA	-	-	-	-
GAA	-	H	-	-
GUA	H	H	-	-
AAA	-	H	-	-
AGA	-	-	-	-
AUA	-	-	-	-
ACA	-	-	-	-
UUA	-	-	-	-
UCA	-	-	-	-
CGA	-	-	-	-
CAA	H	-	-	-
U34 NNGs				
GCG	H	H	-	-
CCG	-	-	-	-
CUG	-	H	-	-
U/C34 NNGs				
GGG	-	-	L	-
GAG	H	H	-	-
GUG	H	H	-	-
AAG	-	-	L	-
AGG	-	-	L	L
AUG	H	H	-	-
ACG	-	H	-	-
UUG	H	H	-	-
UCG	-	H	-	-
CGG	-	H	-	-
CAG	-	-	L	L

* Scored Higher (H) or Lower (L) if A-site codon followed by +1 GNN has significantly higher or lower ribosome densiAes than when not followed by (wo) +1 GNN (bootstrap 2-tailed analysis, $p < 0.01$). The higher (lower) ribosome density is indicaAve of slower (faster) translocaAon rate. Data are for WT1 and WT2 replicate datasets [5].

Table S2: 21-nt RFPs bootstrap analysis (2-tailed; $p < 0.01$)

	+1GNN > wo +1GNN		+1GNN < wo +1GNN*	
	**WT1**	**WT2**	**WT1**	**WT2**
G34 NNUs				
GGU	H	H	-	-
GAU	H	H	-	-
AGU	H	H	-	-
AAU	-	-	-	-
UGU	-	-	-	-
UAU	H	H	-	-
UUU	H	H	-	-
CAU	H	H	-	-
CUU	H	H	-	-
I34 NNUs				
GUU	H	-	-	-
GCU	H	H	-	-
AUU	H	-	-	-
ACU	H	H	-	-
UCU	H	H	-	-
CGU	H	H	-	-
CCU	H	H	-	-
G34 NNCs				
GGC	-	-	L	-
GAC	-	H	-	-
AGC	-	-	-	-
AAC	H	H	-	-
UGC	-	-	-	-
UAC	H	-	-	-
UUC	-	H	-	-
CAC	H	-	-	-
CUC	-	-	-	-
I34 NNCs				
GUC	H	H	-	-
GCC	-	-	-	-
AUC	H	H	-	-
ACC	H	-	-	-
UCC	-	-	-	-
CGC	-	-	-	-
CCC	H	-	-	-

	+1GNN > wo +1GNN		+1GNN < wo +1GNN*	
	WT1	WT2	WT1	WT2
U34 NNAs				
GCA	-	-	-	-
CCA	-	-	-	-
CUA	H	H	-	-
U/C34 NNAs				
GGA	-	-	-	-
GAA	H	H	-	-
GUA	H	-	-	-
AAA	H	H	-	-
AGA	H	H	-	-
AUA	H	-	-	-
ACA	H	H	-	-
UUA	-	-	-	-
UCA	-	-	-	-
CGA	-	-	-	L
CAA	H	H	-	-
U34 NNGs				
GCG	H	H	-	-
CCG	-	-	-	-
CUG	H	H	-	-
U/C34 NNGs				
GGG	-	-	-	-
GAG	H	H	-	-
GUG	H	H	-	-
AAG	H	-	-	-
AGG	-	-	-	L
AUG	H	H	-	-
ACG	-	H	-	-
UUG	H	H	-	-
UCG	-	-	-	-
CGG	-	-	-	-
CAG	H	H	-	-

* Scored Higher (H) or Lower (L) if A-site codon followed by +1 GNN has significantly higher or lower ribosome densities than when not followed by (wo) +1 GNN (bootstrap 2-tailed analysis, $p < 0.01$). The higher (lower) ribosome density is indicative of slower (faster) translocation rate. Data are for WT1 and WT2 replicate datasets [5].

References for supplemental figures and tables

1. Ghaemmaghami, S.; Huh, W.K.; Bower, K.; Howson, R.W.; Belle, A.; Dephoure, N.; O'Shea, E.K.; Weissman, J.S. Global analysis of protein expression in yeast. *Nature* **2003**, *425*, 737-741.
2. Gamble, C.E.; Brule, C.E.; Dean, K.M.; Fields, S.; Grayhack, E.J. Adjacent Codons Act in Concert to Modulate Translation Efficiency in Yeast. *Cell* **2016**, *166*, 679-690, doi:10.1016/j.cell.2016.05.070. PMC4967012
3. Letzring, D.P.; Dean, K.M.; Grayhack, E.J. Control of translation efficiency in yeast by codon-anticodon interactions. *Rna* **2010**, *16*, 2516-2528, doi:10.1261/rna.2411710. PMC2995412
4. Tesina, P.; Lessen, L.N.; Buschauer, R.; Cheng, J.; Wu, C.C.; Berninghausen, O.; Buskirk, A.R.; Becker, T.; Beckmann, R.; Green, R. Molecular mechanism of translational stalling by inhibitory codon combinations and poly(A) tracts. *EMBO J* **2020**, *39*, e103365, doi:10.15252/embj.2019103365. PMC6996574
5. Wu, C.C.; Zinshteyn, B.; Wehner, K.A.; Green, R. High-Resolution Ribosome Profiling Defines Discrete Ribosome Elongation States and Translational Regulation during Cellular Stress. *Mol Cell* **2019**, *73*, 959-970 e955, doi:10.1016/j.molcel.2018.12.009. PMC6411040
